# Voluntary Medical Male Circumcision — Southern and Eastern Africa, 2010–2012

**Published:** 2013-11-29

**Authors:** Mpho Dorothy Seretse, Peter Cherutich, Amon Nkhata, Jotamo Come, Epafras Anyolo, Goitsemodimo Collen Bonnecwe, Gissenge J.I. Lija, Alex Opio, Bushimbwa Tambatamba Chapula, Robert Manda, Samuel Mwalili, Beth A. Tippett Barr, Beverley Cummings, Gram Mutandi, Carlos Toledo, Kokuhumbya J. Kazaura, Monica Dea, Jonas Mwale, Jonathan Grund, Naomi Bock

**Affiliations:** Ministry of Health, Botswana; National AIDS and STI Control Programme, Kenya; Ministry of Health—HIV and AIDS Dept, Malawi; Ministry of Health, Mozambique; Ministry of Health and Social Svcs, Namibia; National Dept of Health, South Africa; Ministry of Health and Social Welfare—National AIDS Control Program, Tanzania; Ministry of Health, Uganda; Ministry of Community Development, Mother and Child Health, Zambia; CDC Botswana; CDC Kenya; CDC Malawi; CDC Mozambique; CDC Namibia; CDC South Africa; CDC Tanzania; CDC Uganda; CDC Zambia; Div of Global HIV/AIDS, Center for Global Health, CDC

Sub-Saharan Africa bears the greatest global burden of human immunodeficiency virus (HIV) infection; 70% (25.0 million) of all persons living with HIV reside in this region (1). Voluntary medical male circumcision (VMMC) has been shown to reduce the risk for heterosexually acquired HIV among men by approximately 60% in three randomized controlled trials (2–5). Further studies found that the protection from HIV acquisition conferred by VMMC was sustained for 6 years following surgery (6,7). In 2007, the World Health Organization (WHO) and Joint United Nations Programme on HIV/AIDS (UNAIDS) recommended that 14 countries with generalized HIV epidemics (i.e., where >1% of the population is HIV-positive) and low male circumcision prevalence* prioritize scale-up of VMMC for HIV prevention (8). On December 1, 2011 (World AIDS Day), funding through the President’s Emergency Plan for AIDS Relief (PEPFAR) was announced to support >4.7 million VMMCs over the next 2 years.† This report presents the results of VMMC scale-up in nine countries where national ministries of health and CDC are implementing VMMC services for HIV prevention: Botswana, Kenya, Malawi, Mozambique, Namibia, South Africa, Tanzania, Uganda, and Zambia. During October 2009–September 2012,§ a total of 1,924,792 VMMCs were performed in 14 countries using PEPFAR funding provided through U.S. government agencies¶; of this total, 1,020,424 were conducted at approximately 1,600 CDC-supported VMMC sites: 137,096 VMMCs in 2010, 347,724 in 2011, and 535,604 in 2012.** Continued program monitoring and quality assurance activities are required to ensure that CDC-supported country programs meet World AIDS Day targets for VMMC.

Data were collected from VMMC client medical forms and country-specific data collection and summarization tools from CDC-supported sites. These data include only VMMCs for HIV prevention, performed under local anesthesia in medical settings by trained clinicians in southern and eastern Africa. All VMMC clients provided informed consent, or assent with permission from a parent or guardian for those aged <18 years. If clinicians determine that a client aged <15 years understands the information provided and is able to cooperate with VMMC under local anesthesia, then surgery can be performed, as long as assent and permission is provided. Data from approximately 1,600 CDC-supported sites were pooled by CDC country offices from local VMMC implementing partners and used to generate summary statistics. Multicountry analyses were conducted to document VMMC progress by examination of data for VMMCs performed, client age, HIV testing and counseling (HTC) acceptance and results, postoperative reviews, and postoperative moderate and severe adverse events (AEs) from 2010–2012. Moderate and severe AEs (e.g., excessive bleeding, infection, swelling, or wound disruption) were classified by type and severity according to PEPFAR’s indicator guidance.†† Some countries use AE definitions that vary slightly from country to country. Annual data were not available from all countries (Table 1).

During 2010–2012, approximately 1,020,424 males were circumcised at CDC-supported sites in the nine countries. The total number of VMMCs has increased each year: 137,096 VMMCs performed in 2010 (seven countries), 347,724 in 2011 (eight countries), and 535,604 in 2012 (nine countries). CDC-supported VMMC programs in Kenya and Uganda performed the most VMMCs during these years: 386,752 and 205,812, respectively (Table 1).

Of the countries reporting data on HTC for VMMC clients (n = 533,143), 86.5% (461,323) of VMMC clients accepted HTC during 2010–2012. Among clients accepting HTC, 2.4% (10,933) tested HIV-positive and were referred to care and treatment services (Table 2). HTC acceptance among VMMC clients varied during this period but remained high: 84.1% in 2010 (four countries), 95.4% in 2011 (five countries), and 83.8% in 2012 (eight countries).

All VMMC clients are advised to return to a health facility for postoperative assessment. Of the countries reporting data on postoperative visits of VMMC clients (n = 614,478), a total of 359,881 clients (58.6%) returned for assessment at the circumcising site within 14 days of surgery. Postoperative follow-up rates have been inconsistent at 75.7% (three countries), 50.0% (five countries), and 64.8% (seven countries) for 2010, 2011, and 2012, respectively. Among all clients returning for postoperative follow-up review within 14 days, the overall postoperative moderate or severe AE rate was low (0.8%), and within acceptable rates for minor surgery. The proportion of clients experiencing a moderate or severe AE has declined from 1.7% in 2010 (three countries) to 0.9% in 2011 (five countries) and 0.8% in 2012 (six countries) (Table 2).

For 986,392 (96.7%) VMMC clients with age reported, the proportion of clients aged ≥15 years increased during 2010–2012. In 2010, the proportion of clients aged ≥15 years was 67.0% (89,280) (six countries), increasing to 78.7% (272,038) (eight countries) in 2011 and 79.4% (400,560) (eight countries) in 2012. The proportion of VMMC clients aged ≥25 years has increased from 0.1% (70) in 2010 (one country), 3.0% (10,249) in 2011 (five countries), and 6.0% (30,553) in 2012 (six countries) (Table 3).

What is already known on this topic?

Voluntary medical male circumcision (VMMC) has been recognized by the World Health Organization and Joint United Nations Programme on HIV/AIDS as an effective human immunodeficiency virus (HIV) prevention intervention in settings with a generalized HIV epidemic and low male circumcision prevalence.

What is added by this report?

This report summarizes progress toward the 2011 World AIDS Day VMMC target of 4.7 million circumcisions by 2013. During 2010–2012, VMMC progress has been increasing in nine countries where CDC supports VMMC service delivery, with 137,096 VMMCs in 2010, 347,724 in 2011, and 535,604 in 2012.

What are the implications for public health practice?

Accelerated VMMC scale-up can be achieved in southern and eastern Africa while maintaining high acceptance of HIV testing and counseling and low rates of adverse events.

## Editorial Note

VMMC is an effective HIV prevention intervention that can be implemented safely in countries in southern and eastern Africa. The announcement on World AIDS Day in 2011 that PEPFAR would support 4.7 million circumcisions provided an achievable goal for VMMC scale-up. In the nine CDC-supported countries, VMMC acceptance has increased nearly fourfold from 2010 to 2012. The postoperative moderate or severe AEs have remained low. Mathematical modeling suggests that reaching 80% VMMC coverage among males aged 15–49 years in the priority countries would require 20.3 million circumcisions by 2015, which would avert approximately 3.4 million HIV infections through 2025 and result in $16.5 billion in net savings from averted HIV care and treatment costs (9).

To reach 80% coverage and the World AIDS Day VMMC goals, country programs have implemented various efficiency models to expedite scale-up. Each of the nine countries included in this analysis has introduced components of WHO’s model for optimizing the volume and efficiency of male circumcision services (i.e., MOVE) (10), including the use of standardized VMMC surgical techniques (nine countries), electrocautery (four countries), use of nonphysicians and lower cadres of health-care providers (nine countries). Most countries rely on nonphysicians (i.e., nurses and clinical officers) to perform VMMC surgery. VMMC country programs are also implementing standardized training programs for all cadres of VMMC providers; targeted, client-specific campaigns to increase demand for VMMC; and routine, site-level quality assurance assessments. Many countries are moving toward a mixed-service delivery model that combines fixed VMMC sites (e.g., permanent sites within existing health-care facilities, such as hospitals and health centers) with mobile and outreach sites (e.g., use of tents, prefabricated structures, and other temporary locations for VMMC service delivery). All sites offering VMMC must provide the “minimum package” of complementary services specified by WHO, including information about the risks and benefits of the procedure, HTC, screening, and treatment of sexually transmitted infections; preoperative and postoperative counseling; and promotion and provision of condoms (10).

In sub-Saharan Africa, men aged 20–39 years are at highest risk for acquiring HIV (1). Only 12.5% (33,420 of 267,158) of VMMC clients during 2010–2012 were aged ≥25 years among those countries reporting this age disaggregation (three countries in 2011 and four in 2012). VMMC programs need to identify innovative approaches to increase VMMC acceptability for men aged ≥25 years. CDC is working in Kenya, Tanzania, and South Africa to evaluate strategies to increase the proportion of older males receiving VMMC and to promote HTC among VMMC clients.

HIV prevalence among adolescents and adults aged 15–49 years of both sexes is high in the nine countries (range: 5.1%–23.0%). Because VMMC clients are all male and generally young (median age: 15–19 years), they would be expected to have a lower HIV prevalence than the general population of persons aged 15–49 years. Among the 461,323 VMMC clients included in this analysis who accepted HTC, 2.4% (10,933) tested HIV-positive (Table 2).

The findings in this report are subject to at least four limitations. First, several countries did not begin scaling up VMMC until 2010 or 2011, which is partially responsible for missing data. Second, because of differing numbers of countries included in the analyses of different variables across years, trends found might not be representative of all VMMC clients. Third, ministry of health–approved client-level data collection tools are not identical across countries, which contributed to difficulties in data aggregation across countries, including the lower age limit for VMMC clients. Finally, some national ministries of health have similar but not identical definitions for classifying type, severity, and clinical signs for VMMC AEs. Although PEPFAR guidance for AE reporting is used in all of PEPFAR’s VMMC programs, discrepant diagnoses and management might result in differences in reporting.

Quality assurance processes should monitor routine reporting of additional VMMC indicators to ensure data availability and to improve data quality. CDC’s external quality assurance activities provide an opportunity to work with ministry of health officials and VMMC implementers to assess and improve data collection and reporting practices. Improved data collection and reporting practices will help CDC-supported country programs meet the World AIDS Day targets for VMMC and achieve an AIDS-free generation.

## Figures and Tables

**TABLE 1:** Voluntary medical male circumcisions (VMMCs) performed by CDC-supported programs, by country and fiscal year, 2010–2013

	No. of VMMCs			
	
Country	2010	2011	2012	Total
Botswana	—	—	8,590	8,590
Kenya*	104,131	166,310	116,311	**386,752**
Malawi†	—	778	7,420	**8,198**
Mozambique	4,009	18,472	68,924	**91,405**
Namibia	1,197	5,292	5,965	**12,454**
South Africa§	3,820	15,574	80,701	**100,095**
Tanzania¶	1,519	50,325	49,756	**101,600**
Uganda	9,052	57,132	139,628	**205,812**
Zambia	13,368	33,841	58,309	**105,518**
**Total**	**137,096**	**347,724**	**535,604**	**1,020,424**

**Source:** President’s Emergency Plan for AIDS Relief (PEPFAR) annual progress report (APR) submissions for CDC-supported partners, for fiscal years October 1–September 30, except where noted.

* Kenya’s data for 2010 and 2011 are reported from January–December, but data from 2012 are from October-September.

† Malawi’s data are from APR results and CDC Malawi’s partner reports for 2012.

§ South Africa’s data are reported from January–December for 2010–2012.

¶ Tanzania’s data for 2010–2012 are from APR reports and Tanzania’s national database.

**TABLE 2:** Voluntary medical male circumcision (VMMC) progress, HIV testing and counseling (HTC) acceptance, human immunodeficiency virus (HIV) prevalence among VMMC clients, postoperative follow-up reviews among VMMC clients, and postoperative moderate or severe VMMC adverse event (AE) rates, by country and year, 2010–2012

	Total VMMCs performed	HTC uptake among VMMC clients		HIV prevalence among VMMC clients		Postoperative follow-up within 14 days of VMMC		Postoperative moderate or severe AEs	
					
Country	No.	No.	(%)	No.	(%)	No.	(%)	No.	(%)
**2012**									
Botswana	8,590	7,702	(89.7)	234	(3.0)	6,571	(76.5)	136	(2.1)
Kenya	116,311	97,647	(84.0)	3,386	(3.5)	40,084	(34.5)	506*	(1.3)
Malawi	7,420	526	(53.1)	38	(7.2)	398	(40.2)	—	—
Mozambique	68,924	68,924	(100.0)	1,915	(1.3)	—	—	—	—
Namibia	5,965	5,259	(88.2)	69	(1.3)	5,777	(96.8)	62	(1.1)
South Africa	80,701	79,087	(98.0)	1,788	(2.3)	72,631	(90.0)	290	(0.4)
Tanzania†	49,756	43,637	(87.7)	590	(1.4)	41,561	(83.5)	272*	(0.7)
Uganda	139,628	—	—	—	—	—	—	—	—
Zambia	58,309	23,802	(40.8)	165	(0.7)	45,026	(77.2)	351*	(0.8)
**Total**	**535,604**	**326,584**	**(83.8)**	**8,185**	**(2.5)**	**212,048**	**(64.8)**	**1,617**	**(0.8)**
**2011**									
Botswana	—	—	—	—	—	—	—	—	—
Kenya	166,310	—	—	—	—	42,937	(25.8)	284*	(0.7)
Malawi	778	—	—	—	—	—	—	—	—
Mozambique	18,472	18,472	(100.0)	753	(4.1)	—	—	—	—
Namibia	5,292	4,770	(90.1)	71	(1.5)	5,084	(96.1)	92	(1.8)
South Africa	15,574	13,091	(84.1)	526	(4.0)	12,023	(77.2)	87	(0.7)
Tanzania†	50,325	47,658	(94.8)	636	(1.3)	48,078	(95.5)	421*	(0.9)
Uganda	57,132				—	—	—	—	—
Zambia	33,841	33,841	(100.0)	339	(1.0)	27,530	(81.4)	252	(0.9)
**Total**	**347,724**	**117,832**	**(95.4)**	**2,325**	**(2.0)**	**135,652**	**(50.0)**	**1,196**	**(0.9)**
**2010**									
Botswana	—	—	—	—	—	—	—	—	—
Kenya	104,131	—	—	—	—	—	—	—	—
Malawi	—	—	—	—	—	—	—	—	—
Mozambique	4,009	3,701	(92.3)	154	(4.2)	—	—	—	—
Namibia	1,197	996	(83.2)	26	(2.6)	891	(74.4)	20	(2.2)
South Africa	3,820	—	—	—	—	—	—	—	—
Tanzania†	1,519	1,346	(88.6)	16	(1.2)	1,267	(83.4)	53*	(4.2)
Uganda	9,052	—	—	—	—	—	—	—	—
Zambia	13,368	10,864	(81.3)	227	(2.1)	10,023	(75.0)	130	(1.3)
**Total**	**137,096**	**16,907**	**(84.1)**	**423**	**(2.5)**	**12,181**	**(75.7)**	**203**	**(1.7)**
**Summary 2010–2012**									
Botswana	8,590	7,702	(89.7)	234	(3.0)	6,571	(76.5)	136	(2.1)
Kenya	386,752	97,647	(84.0)	3,386	(3.5)	—	—	790	(—)
Malawi	8,198	526	(53.1)	38	(7.2)	398	(5.4)	—	(—)
Mozambique	91,405	91,097	(99.7)	2,822	(3.1)	—	—	446	(—)
Namibia	12,454	11,025	(88.5)	166	(1.5)	11,752	(94.4)	174	(1.5)
South Africa	100,095	92,178	(95.7)	2,314	(2.5)	84,654	(87.9)	377	(0.4)
Tanzania†	101,600	92,641	(91.2)	1,242	(1.3)	90,906	(89.5)	746	(0.8)
Uganda	205,812	—	—	—	—	—	—	470	(—)
Zambia	105,518	68,507	(64.9)	731	(1.1)	82,579	(78.3)	733	(0.9)
**Total**	**1,020,424**	**461,323**	**(86.5)**	**10,933**	**(2.4)**	**359,881**	**(58.6)**	**3,016**	**(0.8)**

* Contains both intraoperative and moderate or severe postoperative AEs.

† Tanzania’s data for postoperative follow-up visits are within 48 hours of surgery, not 14 days. Tanzania’s national database collects HTC data on all patients regardless of whether they received VMMC. HTC acceptance among VMMC clients in this table has been imputed by using HTC data from all clients testing at the VMMC site.

**TABLE 3:** Voluntary medical male circumcisions, by age group, country, and year, 2010–2012

	2012					
	
	Age group (yrs)					
	
Country	<15	≥15	15–19	20–24	≥25	Total
Botswana	865	7,725	2,385	2,267	3,073	**8,590**
Kenya*	16,725	99,586	99,586	—	—	**116,311**
Malawi	—	—	—	—	—	**—**
Mozambique*	36,504	32,420	20,388	6,988	5,044	**68,924**
Namibia	1,183	4,782	4,782	—	—	**5,965**
South Africa	11,825	68,876	37,069	16,738	15,069	**80,701**
Tanzania†	24,209	25,547	22,139	—	3,408	**49,756**
Uganda*,§	43,540	90,549	90,549	—	—	**134,089**
Zambia	13,121	30,150	23,518	2,673	3,959	**43,271**
**Total**	**147,972**	**400,560**	**300,416**	**28,666**	**30,553**	**507,607**
Percentage	29.2%	79.4%	59.2%	5.6%	6.0%	**100.0%**

	**2011**					
	
	**Age group (yrs)**					
	
**Country**	**<15**	**≥15**	**15–19**	**20–24**	**≥25**	**Total**

Botswana	—	—	—	—	—	**—**
Kenya*	20,129	146,181	146,181	—	—	**166,310**
Malawi*	180	598	598	—	—	**778**
Mozambique*	7,181	11,291	5,958	3,185	2,148	**18,472**
Namibia*	976	4,316	4,316	—	—	**5,292**
South Africa§	295	13,064	6,467	3,651	2,946	**13,359**
Tanzania†	19,432	30,893	26,919	—	3,974	**50,325**
Uganda*	16,406	40,726	40,726	—	—	**57,132**
Zambia	8,872	24,969	20,139	3,649	1,181	**33,841**
**Total**	**73,471**	**272,038**	**251,304**	**10,485**	**10,249**	**345,509**
Percentage	21.3%	78.7%	72.7%	3.0%	3.0%	**100.0%**

	**2010**					
	
	**Age group (yrs)**					
	
**Country**	**<15**	**≥15**	**15–19**	**20–24**	**≥25**	**Total**

Botswana	—	—	—	—	—	**—**
Kenya*	36,565	67,566	67,566	—	—	**104,131**
Malawi	—	—	—	—	—	**—**
Mozambique*	492	3,517	3,517	—	—	**4,009**
Namibia*	28	1,169	1,169	—	—	**1,197**
South Africa	—	—	—	—	—	**—**
Tanzania†	417	1,102	1,032	—	70	**1,519**
Uganda*	2,561	6,491	6,491	—	—	**9,052**
Zambia	3,933	9,435	9,435	—	—	**13,368**
**Total**	**43,969**	**89,280**	**89,210**	**—**	**70**	**133,276**
Percentage	33.0%	67.0%	66.9%	0.0%	0.1%	**100.0%**

**Country**	**Summary 2010–2012**					
	
	**Age group (yrs)**					
	
	**<15**	**≥15**	**15–19**	**20–24**	**≥25**	**Total**

Botswana	865	7,725	2,385	2,267	3,073	**8,590**
Kenya*	73,419	313,333	313,333	0	0	**386,752**
Malawi	180	598	598	0	0	**778**
Mozambique*	44,177	47,228	29,863	10,173	7,192	**91,405**
Namibia*	2,187	10,267	10,267	0	0	**12,454**
South Africa	12,120	81,940	43,536	20,389	18,015	**94,060**
Tanzania†	44,058	57,542	50,090	0	7,452	**101,600**
Uganda*	62,507	137,766	137,766	0	0	**200,273**
Zambia	25,926	64,554	53,092	6,322	5,140	**90,480**
**Total**	**265,439**	**761,878**	**640,930**	**39,151**	**40,872**	**986,392**
Percentage	26.9%	77.2%	65.0%	4.0%	4.1%	**100.0%**

* These countries only reported age groups as 1–14 years and ≥15 years.

† Tanzania’s age groups reported as <15, 15–25, and ≥26 years.

§ Age missing for some VMMC clients.

## Footnotes

* The 14 countries with 2013 HIV prevalence reported include Botswana (23.0%), Ethiopia (1.3%), Kenya (6.1%), Lesotho (23.1%), Malawi (10.8%), Mozambique (11.1%), Namibia (13.3%), Rwanda (2.9%), South Africa (17.9%), Swaziland (26.5%), Tanzania (5.1%), Uganda (7.2%), Zambia (12.7%), and Zimbabwe (14.7%).

† Additional information available at http://www.whitehouse.gov/the-press-office/2011/12/01/fact-sheet-beginning-end-aids.

§ Data are reported by fiscal year in this report, unless noted otherwise. U.S. government fiscal year is October 1–September 30.

¶ Summary results from PEPFAR’s 2012 annual progress report are available at http://www.pepfar.gov/documents/organization/201387.pdf.

** CDC support includes hiring of clinical staff to provide VMMCs, conducting trainings and quality assurance assessments, providing technical assistance, and procurement of VMMC supplies, medications, and instruments.

†† PEPFAR’s indicator reference sheet for VMMC is available at http://www.malecircumcision.org/resources/documents/PEPFAR_Guide_Monitoring_Reporting_VMMC_Indicators_Appendices.pdf.
